# Digital Information Sharing Before Consultations in General Practice: Protocol for a Scoping Review

**DOI:** 10.2196/82649

**Published:** 2025-12-03

**Authors:** Mohammad S Razai, Mikail Khawaja, Zahir Shah, Isla Kuhn, Hajira Dambha-Miller, Pippa Oakeshott, Simon J Griffin

**Affiliations:** 1 Primary Care Unit, Department of Public Health and Primary Care University of Cambridge Cambridge United Kingdom; 2 School of Clinical Medicine University of Cambridge Cambridge, England United Kingdom; 3 Primary Care Research Centre, Aldermoor Health Centre University of Southampton Southampton United Kingdom; 4 St George’s School of Health and Medical Sciences, Population Health Research Institute City St George's University of London London United Kingdom

**Keywords:** digital health, online consultation, preconsultation information sharing, asynchronous communication, Accurx, eConsult, general practice, telehealth, primary care, scoping review, artificial intelligence, AI

## Abstract

**Background:**

Digital tools that enable patients to submit information before consultations, such as Accurx and eConsult, are increasingly used in general practice. These systems aim to streamline workflows, improve documentation, and optimize consultation efficiency. However, evidence about their implementation, impact on health inequalities, and health care outcomes remains limited and fragmented.

**Objective:**

This study aims to map and synthesize the evidence on digital tools used for preconsultation information sharing in family or general practice.

**Methods:**

This scoping review will follow the Joanna Briggs Institute framework and the PRISMA-ScR (Preferred Reporting Items for Systematic Reviews and Meta-Analyses Extension for Scoping Reviews) guidelines. Searches were conducted on May 12, 2025, in MEDLINE (Ovid), Embase (Ovid), CINAHL (EBSCOhost), and the Cochrane Library. Gray literature will be identified via Google Scholar and the National Health Service or government websites. Eligible studies will describe or evaluate digital tools used to collect information from patients before general practice consultations. Two independent reviewers will conduct screening and data extraction. Data will be analyzed using narrative synthesis.

**Results:**

Database searches identified 6991 records, with 4536 (64.88%) remaining after deduplication. Screening began in June 2025. Full-text screening was completed in November 2025, with data extraction and synthesis planned for completion by February 2026. Results will be submitted for publication in early 2026.

**Conclusions:**

This review will summarize evidence concerning the use of digital tools for preconsultation information sharing in general practice. Findings will inform implementation, research priorities, and service improvement in digitally supported care.

**International Registered Report Identifier (IRRID):**

DERR1-10.2196/82649

## Introduction

### Rationale

Digital tools that enable patients to share information before health care consultations have become increasingly common, particularly in general practice. Systems, such as Accurx, allow patients to submit descriptions of symptoms, photographs, or queries, facilitating asynchronous communication between patients and clinicians [1,2].

Before the COVID-19 pandemic, online consultation systems were already in place in the United Kingdom and elsewhere [3-5]. These typically involved patients submitting online forms that described their clinical or administrative needs, which were reviewed by health care staff who resolved the issue directly or arranged a follow-up via telephone, online messaging, or a face-to-face consultation. Early studies, predominantly from the United Kingdom, focused on implementation, user experience, and the potential of these systems to increase efficiency and manage workload.

A recent systematic review of studies up to 2022 examined asynchronous telemedicine (also known as the “store-and-forward” technique, where clinical data, such as text or images, are transmitted and interpreted later) in general practice. It found that asynchronous telemedicine can support effective diagnosis, prescribing, and timely care, with increased use after the COVID-19 pandemic. However, evidence remains limited by heterogeneity and small study sizes, with gaps in reporting on safety, equity, and cost-effectiveness [6]. There are also concerns that digital exclusion may exacerbate health inequalities.

Since the COVID-19 pandemic, there has been a substantial acceleration in the adoption of digital technologies across primary care, including increased use of artificial intelligence–enabled tools to support triage, decision-making, and patient communication. Digital transformation is also a priority for improving care and reducing costs, as outlined in the UK government’s digital health and social care plan [7]. In particular, the National Health Service (NHS) aims to “put digital tools in place so patients can be supported with high-quality information that equips them to take greater control over their health and care” [8].

At the same time, the structure and delivery of general practice have changed, now offering a wider and more complex range of services provided by an expanded multidisciplinary team. This includes incorporating allied health professionals, such as physiotherapists, pharmacists, paramedics, and physician assistants (formerly known as physician associates), reflecting a move toward team-based, digitally supported models of care.

While these technologies aim to improve efficiency and patient experience, the breadth, mechanisms, socioeconomic impacts, and health outcomes of such digital tools remain underexplored [9]. Therefore, we aim to map the available evidence on digital preconsultation information sharing in general practice settings.

### Objectives

This scoping review will follow the PRISMA-ScR (Preferred Reporting Items for Systematic Reviews and Meta-Analyses Extension for Scoping Reviews) guidelines [10] (Multimedia Appendix 1). The objectives are to (1) identify and map digital methods used for information sharing before telephone, virtual, and face-to-face consultations in general practice; (2) describe the characteristics, implementation contexts, and reported impacts and outcomes of such systems; (3) explore the range of digital tools (eg, Accurx and eConsult) used; and (4) identify gaps in the current literature that require further empirical research or systematic review.

### Review Questions

This review will be guided by the following questions, using the Joanna Briggs Institute population, concept, and context framework [11]:

- What digital tools (concept) are used to collect information before health care consultations in general practice (context)?
- In which patient populations (population) are these tools implemented?
- What are the reported experiences and outcomes (concept) of using these tools in general practice (context)?
- What methodological approaches have been used to evaluate their implementation or effectiveness (concept) in general practice (context)?
- What is the evidence on the effects on health inequalities and health outcomes (context)?

## Methods

### Eligibility Criteria

Studies will be selected according to predefined inclusion and exclusion criteria (Textbox 1), designed to identify relevant literature on digital tools used for information sharing before consultations in general practice.

**Inclusion and exclusion criteria.**

**Inclusion criteria**

- Studies describing or evaluating digital, online, or virtual tools used to gather information before consultations (eg, face-to-face, telephone, or video)
- Tools combining patient-entered data with algorithmic triage (eg, eConsult, askmyGP, and Accurx), provided they allow patients to submit information before clinician review
- Studies conducted in family or general practice settings
- All study designs, including qualitative, quantitative, and mixed methods
- Publications in English
- Studies from any country

**Exclusion criteria**

- Synchronous telemedicine, including remote telephone and virtual consultations (audio only or video consultations)
- Studies in primary and community settings, which are not part of family or general practice (eg, community nursing, pharmacy, dental care, optometry, and mental health services), with the context of interest being general practice, including general practitioner–led teams but excluding independent community or pharmacy-only services
- Studies focused only on remote consultations without a preconsultation component
- Tools limited to automated decision support (eg, back-end artificial intelligence decision aids used only by clinicians) without patient input
- Telemonitoring interventions
- Interactions solely between health care professionals
- Editorials, commentaries, opinion pieces, study protocols, case reports, conference abstracts, research theses, and policy documents

### Information Sources and Search Strategy

A structured literature search was conducted on May 12, 2025, across 4 electronic databases: MEDLINE (via Ovid), Embase (via Ovid), CINAHL (via EBSCOhost), and the Cochrane Library. Searches were limited to studies published from January 2021 onward to reflect the rapid evolution of digital tools in general practice. The strategy combined terms related to general practice (eg, “primary care,” “general practitioner,” and “family medicine”) with terms describing digital and asynchronous consultation methods (eg, “Accurx,” “eConsult,” “online consultation,” “remote consultation,” and “telemedicine”).

Gray literature and policy documents will be identified through targeted searches of Google Scholar and relevant NHS or government websites. The database searches yielded 6991 records, with 4536 (64.88%) remaining after deduplication. The complete search strategy is provided in Multimedia Appendix 2.

The search was restricted to studies from January 2021 onward to capture post–COVID-19 pandemic acceleration of digital preconsultation systems. Earlier literature has been summarized in previous reviews of online consultation tools conducted before or during early COVID-19 phases [3-6]. As a sensitivity check, we will screen a sample of highly cited pre-2021 records to confirm no new concepts have been omitted. Non-English studies with English abstracts will be considered at the abstract level.

### Selection of Sources of Evidence

Three researchers will screen all titles and abstracts against the eligibility criteria. There will be an initial pilot phase to operationalize and standardize definitions to optimize levels of agreement. To ensure consistency, an initial 20% of the titles and abstracts will be independently screened in duplicate to calibrate reviewers and estimate interrater agreement (target Cohen κ≥0.8) [12]. If the agreement falls below this threshold, the proportion screened in duplicate will be increased. Screening will be managed in Rayyan [13], with automated deduplication and tracking via EndNote (Clarivate). All full-text articles will undergo duplicate screening. Any discrepancies will be resolved through discussion or adjudication by a third reviewer.

### Definition of Key Concepts

*General practice* is the first point of contact in the health care system. It provides a range of services, including diagnosing and treating medical conditions, managing long-term illnesses, prescribing medicines, delivering vaccinations, promoting health, and referring patients for specialist care. General practice includes general practitioners (GPs), nurses, allied health professionals (eg, pharmacists, physician assistants, and physiotherapists), and administrative staff working as part of a multidisciplinary team [14].

*Consultation* is defined based on a theoretical framework derived from the model of the patient-centered consultation proposed by Stewart et al [15]. This model provides the most widely cited definition of consultation in UK primary care. It encompasses the following elements: (1) exploring both the disease and the illness experience; (2) understanding the whole person; (3) finding common ground regarding management; (4) incorporating prevention and health promotion; (5) strengthening the physician-patient relationship; and (6) “being realistic” and recognizing personal limitations and practical constraints, such as the availability of time and resources

*Digital* refers to “the use and transfer of information using devices and technology, such as computers and smartphone applications, and the infrastructure and processes used to do so” [16].

*Telemedicine* “is the use of telecommunication and information technology for the purpose of providing remote health assessments and therapeutic interventions” [17].

*Synchronous telehealth* refers to real-time communication between a patient and a health care professional in different locations, using audio or video technology [18].

*Asynchronous telehealth* refers to the use of digital tools that enable the collection and review of clinical information, such as text, images, or data, at a later time. This includes store-and-forward systems as well as online patient queries submitted via portals or apps [18]. This is a communication where clinician review occurs later and not in real time.

*Preconsultation* information sharing refers to any digital submission of information that precedes synchronous clinical interaction.

*Demographic factors* refer to the characteristics of populations that help describe who is using digital tools and how use may vary. In this review, demographic factors include age and sex or gender.

*Socioeconomic factors* refer to an individual’s social and economic position, which may influence access to and engagement with digital health services.

The following definitions apply:

- Age refers to the patient’s chronological age, which may affect digital literacy, health needs, and engagement with technology.
- Gender refers to socially constructed roles and identities (eg, male, female, or nonbinary), which can influence health-seeking behavior and communication preferences.
- Education level reflects the highest level of formal education completed and may affect digital competence and health literacy.
- Income level refers to an individual’s or household’s financial resources, which can shape access to technology, internet connectivity, and health services.

### Data Collection Process

Data will be extracted using a prepiloted data charting form developed by the review team. There will be an initial pilot phase using the form to operationalize and standardize definitions and optimize levels of agreement. Two reviewers will independently extract data from all included studies. Discrepancies will be resolved through discussion or by a third reviewer, if needed. Extracted data will include authors, year, country, study design, health care setting, description of the digital tool or system, target population, implementation features, outcomes measured, and key findings. Extracted data will be cross-checked against predefined review questions and outcome categories to ensure consistency and accuracy.

### Synthesis of Results

Extracted data will be summarized in tabular and narrative form. Thematic analysis will be used for qualitative data, and frequency counts will be used to summarize key characteristics across studies. Qualitative findings will be organized using a deductive framework derived from established domains in digital health evaluations: access and equity, workload or substitution, timeliness, safety, usability, costs, and implementation context. A preliminary codebook will be piloted on 10 studies and refined iteratively. Quantitative outcomes will be tabulated descriptively by study design and population subgroup (eg, age, deprivation, ethnicity, rurality, and digital exclusion).

### Quality Appraisal and Risk of Bias Assessment

Although formal quality assessment is not mandatory in scoping reviews, we will use the Mixed Methods Appraisal Tool (MMAT; version 2018, Canadian Intellectual Property Office) to assess the methodological quality of included studies. The MMAT is designed to appraise qualitative, quantitative, and mixed methods studies within a single tool, making it well suited to the diverse evidence base anticipated in this review. Given the likelihood of heterogeneity in study designs, the MMAT provides a consistent and structured approach to assess study rigor (without excluding studies based on quality) and supports transparent reporting of study limitations [19]. Two reviewers will independently assess the methodological quality of all included studies using the MMAT [19]. Disagreements will be resolved through discussion or by a third reviewer, if needed.

### Dissemination

Findings will be disseminated in a peer-reviewed journal and at an academic conference. The results may inform future implementation strategies and areas for targeted evaluation.

## Results

This scoping review began in May 2025. Full-text screening and final eligibility assessments were completed in November 2025. Data extraction and analysis will be conducted between December 2025 and February 2026, with findings to be submitted for publication in a peer-reviewed journal in early 2026.

**Figure 1:**
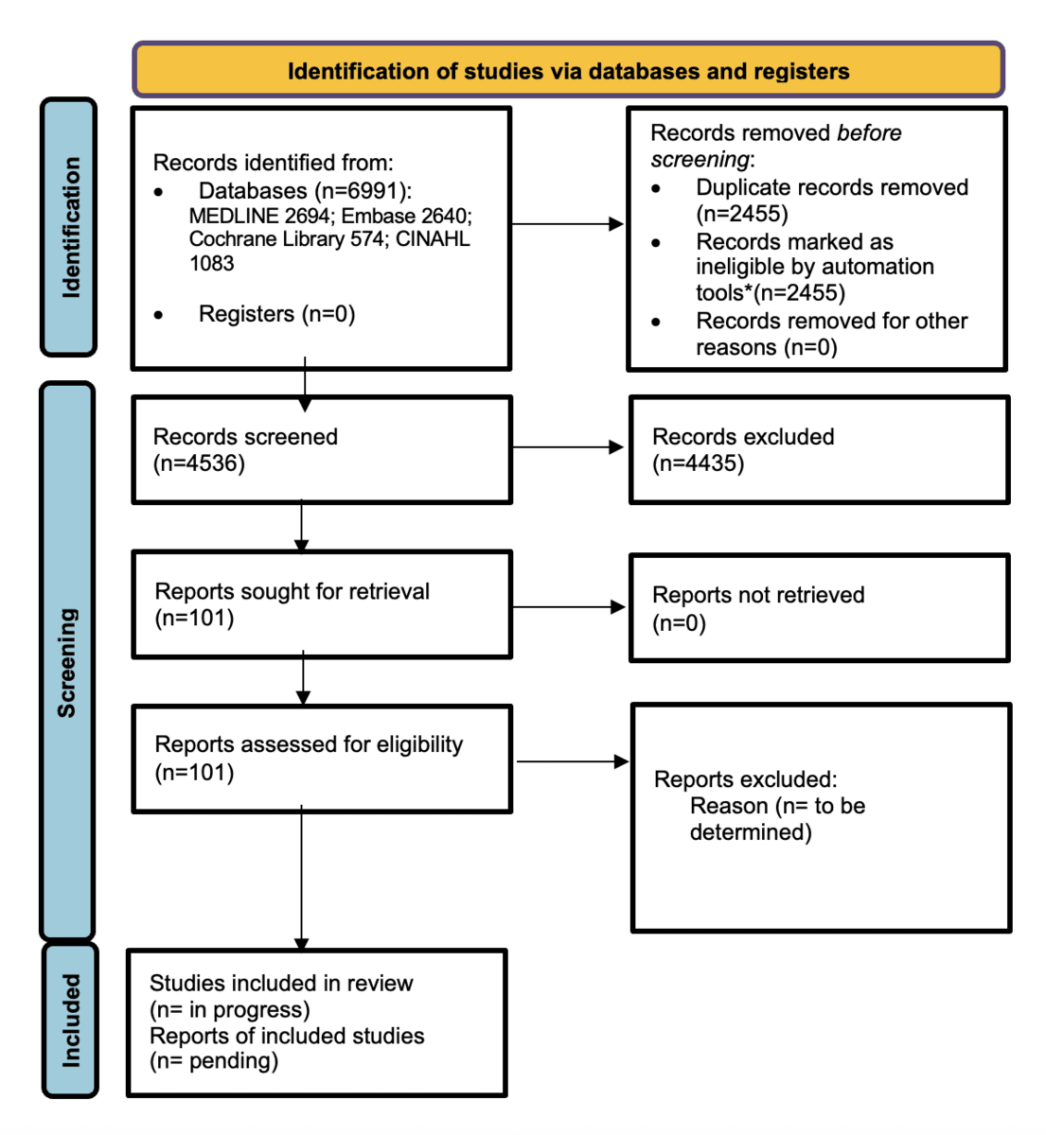
Preliminary PRISMA flow diagram. *Automation tools: Rayyan and EndNote (Clarivate).

On the basis of previous research, we anticipate considerable heterogeneity in study design, populations, and outcome measures. However, more recent and larger-scale evaluations are also likely to be included, reflecting the increasing adoption of asynchronous digital information-sharing tools in primary care. This review aims to improve understanding of how patients share information digitally before consultations and the implications for quality, safety, equity, and clinical outcomes in general practice. Figure 1 summarizes the study selection process using the PRISMA (Preferred Reporting Items for Systematic Reviews and Meta-Analyses) flow diagram [20].

## Discussion

### Anticipated Findings

This review will summarize evidence concerning the use of digital tools for preconsultation information sharing in general practice. Findings will inform implementation, research priorities, and service improvement in digitally supported care and could impact health inequalities.

### Strengths and Limitations

This review will be the first scoping review to systematically map the evidence on digital tools used for preconsultation information sharing specifically within general practice, following their widespread adoption in the wake of the COVID-19 pandemic. It will use a comprehensive search strategy across multiple databases; follow the PRISMA-ScR guidelines; and apply the Joanna Briggs Institute population, concept, and context framework to guide study selection. The inclusion of all study designs and the use of MMAT will allow for consistent assessment of methodological quality across heterogeneous evidence.

However, as a scoping review, it will not include a meta-analysis. Quality appraisal will support interpretation but not be used to exclude studies. In addition, the focus on general practice may limit the generalizability of findings to other health care settings. Partial duplicate screening risks missing eligible studies. However, our approach involves an initial 20% dual screening with interrater reliability checks (target κ≥0.8) and verification by a third reviewer when discrepancies arise.

### Comparison With Other Studies

A range of digital tools has been introduced in general practice to allow patients to share information before consultations. A retrospective evaluation in Spain found that eConsulta (an asynchronous teleconsultation tool) could avoid in-person appointments in up to 87.9% of cases, particularly for test results, medical queries, and repeat prescriptions. However, 27.7% of interactions represented additional demand, potentially stimulated by ease of access [4].

In England, similar platforms (eg, webGP, askmyGP, Tele-Doc, and eConsult) have been piloted and studied with mixed outcomes before the COVID-19 pandemic. A mixed methods evaluation of webGP piloted across 6 practices in Devon revealed low uptake, with GPs judging that 72% of the online requests still required a telephone or face-to-face consultation. While patients found the system broadly acceptable, it shifted responsibilities between patients and practice staff, introducing tensions with existing workflows [3]. A retrospective study analyzing data from 9 UK practices using askmyGP found that use was highest among female individuals and those aged between 25 and 34 years. Online activity peaked early in the week and during morning hours. Most consultations were for medication-related or administrative reasons. While some patients reported convenience and ease of use, these benefits were context dependent [5].

Other studies highlighted implementation challenges. For example, Tele-Doc uptake was limited, with increased responsibility placed on patients and administrative staff [21]. A 15-month pilot across 36 practices, uptake of eConsult was low (mean 2 consultations per 1000 patients per month), with most consultations occurring during working hours. Most patients were women (64.7%), with a median age of 39 years. The most common reasons were administrative requests and infections. Approximately 70% of the patients received a follow-up via telephone or face-to-face, and the average cost per e-consultation response was £36.28 (about US $49.20), mainly driven by subsequent face-to-face or telephone consultations [22].

A sequential mixed methods evaluation of eConsult use in Devon and Cornwall found that while COVID-19 led to increased use, concerns remained about usability, repetitive forms, and the lack of continuity. Older adults had lower uptake despite being frequent users of face-to-face care. Only 3% of the consultations included feedback forms, and GP websites often failed to meet accessibility standards [23]. A 6-month evaluation across 11 practices in Scotland found that eConsult largely met expectations as an additional access route to GP services but there was less certainty in its ability to promote self-management. Uptake was low, and successful implementation required strong internal leadership and process protocols [24].

An analysis of NHS clinical commissioning groups’ annual reports in England revealed wide variation in digital capabilities across regions [25]. Using a digital options framework, 3 clusters of digital maturity were identified: digitally disengaged, digitally engaged, and digital torchbearers. Despite national policies promoting digital transformation, almost half of clinical commissioning groups, particularly in London, were classified as digitally disengaged. The study suggested that improving digital health literacy and inclusion efforts is as important as advancing technical innovations [25]. Evidence from the Netherlands also suggests that digital tools may increase overall consultation rates and associated costs, potentially reflecting greater accessibility [26].

A systematic review using normalization process theory identified key factors influencing the successful implementation of asynchronous online platforms. Although patients valued the convenience of digital consultations and health care staff felt confident using the platforms, integration issues, increased workload, usability problems, and concerns about confidentiality and health inequities prevented embedding into routine practice. Recommendations included improving platform usability, integration with clinical systems, and targeted support for different patient groups [27]. In addition, digital preconsultation systems may inadvertently widen access gaps. Patients with limited English proficiency, low health literacy, or limited digital access could find these platforms more difficult to use, leading to differential engagement and outcomes. Understanding how such tools affect equity of access will be a critical dimension of future evaluation.

### Implications for Practice and Research

This review will offer a comprehensive synthesis of how digital tools are used to collect patient information before consultations in general practice. Findings will inform clinicians, commissioners, and policymakers about the range and characteristics of preconsultation digital tools, their implementation contexts, and reported outcomes. This may guide more effective integration of asynchronous digital tools into general practice workflows and highlight implications for workload, access, and patient experience. The review will also identify evidence gaps and inform priorities for future evaluation and implementation research, particularly about safety, equity, and system-wide adoption.

### Conclusions

Asynchronous digital tools for preconsultation information sharing are becoming increasingly integrated into general practice. However, the evidence on their design, use, and impact on health inequalities remains fragmented. This review aims to map the existing literature and provide a foundation for future research and implementation strategies that support the safe, equitable, and effective use of digital tools in general practice.

## Data Availability

Data are supplied in supporting files available for download along with the published manuscript.

## Appendix

Multimedia Appendix 1 PRISMA-ScR checklist.

Multimedia Appendix 2 Complete search strategy.

## Abbreviations

GP: general practitioner
MMAT: Mixed Methods Appraisal Tool
NHS: National Health Service
PRISMA: Preferred Reporting Items for Systematic Reviews and Meta-Analyses
PRISMA-ScR: Preferred Reporting Items for Systematic Reviews and Meta-Analyses Extension for Scoping Reviews

## References

[1] Mittal R, Kannan AK, Mohindroo R, Movva C, Zhang L, Reehana S, Srinivasan S (2025). Implementing accurx for total triage enhancing care navigation and patient experience. Cureus.

[2] Patient Triage: what information do patients submit?. Accurx.

[3] Carter M, Fletcher E, Sansom A, Warren FC, Campbell JL (2018). Feasibility, acceptability and effectiveness of an online alternative to face-to-face consultation in general practice: a mixed-methods study of webGP in six Devon practices. BMJ Open.

[4] López Seguí F, Vidal-Alaball J, Sagarra Castro M, García-Altés A, García Cuyàs F (2020). General practitioners' perceptions of whether teleconsultations reduce the number of face-to-face visits in the Catalan public primary care system: retrospective cross-sectional study. J Med Internet Res.

[5] Eccles A, Hopper M, Turk A, Atherton H (2019). Patient use of an online triage platform: a mixed-methods retrospective exploration in UK primary care. Br J Gen Pract.

[6] Leighton C, Cooper A, Porter A, Edwards A, Joseph-Williams N (2024). Effectiveness and safety of asynchronous telemedicine consultations in general practice: a systematic review. BJGP Open.

[7] A plan for digital health and social care. Goverment of UK.

[8] 2023/24 priorities and operational planning guidance. National Health Service (NHS) England.

[9] Razai MS, Dambha-Miller H, Griffin S (2025). Digital platforms in primary care: leveraging asynchronous consultations to support management of cardiometabolic diseases and risk factors. J Prim Care Community Health.

[10] Tricco AC, Lillie E, Zarin W, O'Brien KK, Colquhoun H, Levac D, Moher D, Peters MD, Horsley T, Weeks L, Hempel S, Akl EA, Chang C, McGowan J, Stewart L, Hartling L, Aldcroft A, Wilson MG, Garritty C, Lewin S, Godfrey CM, Macdonald MT, Langlois EV, Soares-Weiser K, Moriarty J, Clifford T, Tunçalp Ö, Straus SE (2018). PRISMA extension for scoping reviews (PRISMA-ScR): checklist and explanation. Ann Intern Med.

[11] Joanna Briggs Institute (2014). Joanna Briggs Institute Reviewers’ Manual: 2014 edition.

[12] Landis JR, Koch GG (1977). The measurement of observer agreement for categorical data. Biometrics.

[13] Ouzzani M, Hammady H, Fedorowicz Z, Elmagarmid A (2016). Rayyan-a web and mobile app for systematic reviews. Syst Rev.

[14] General practice in England. UK Parliament.

[15] Stewart M, Brown JB, Weston WW, Freeman T, Ryan BL, McWilliam C, McWhinney IR (2014). Patient-Centered Medicine: Transforming the Clinical Method.

[16] Digital tools for online consultation in general practice. The Health Services Safety Investigations Body (HSSIB).

[17] Telemedicine 2022. NHS England.

[18] (2021). Telehealth resource center: definitions. American Medical Association.

[19] Hong QN, Fàbregues S, Bartlett G, Boardman F, Cargo M, Dagenais P, Gagnon M, Griffiths F, Nicolau B, O’Cathain A, Rousseau M, Vedel I, Pluye P (2018). The Mixed Methods Appraisal Tool (MMAT) version 2018 for information professionals and researchers. Educ Inf.

[20] Page MJ, McKenzie JE, Bossuyt PM, Boutron I, Hoffmann TC, Mulrow C, Shamseer L, Tetzlaff JM, Akl EA, Brennan SE, Chou R, Glanville J, Grimshaw JM, Hróbjartsson A, Lalu MM, Li T, Loder EW, Mayo-Wilson E, McDonald S, McGuinness LA, Stewart LA, Thomas J, Tricco AC, Welch VA, Whiting P, Moher D (2021). The PRISMA 2020 statement: an updated guideline for reporting systematic reviews. BMJ.

[21] Casey M, Shaw S, Swinglehurst D (2017). Experiences with online consultation systems in primary care: case study of one early adopter site. Br J Gen Pract.

[22] Edwards HB, Marques E, Hollingworth W, Horwood J, Farr M, Bernard E, Salisbury C, Northstone K (2017). Use of a primary care online consultation system, by whom, when and why: evaluation of a pilot observational study in 36 general practices in South West England. BMJ Open.

[23] Jones RB, Tredinnick-Rowe J, Baines R, Maramba ID, Chatterjee A (2022). Use and usability of GP online services: a mixed-methods sequential study, before and during the COVID-19 pandemic, based on qualitative interviews, analysis of routine eConsult usage and feedback data, and assessment of GP websites in Devon and Cornwall, England. BMJ Open.

[24] Cowie J, Calveley E, Bowers G, Bowers J (2018). Evaluation of a digital consultation and self-care advice tool in primary care: a multi-methods study. Int J Environ Res Public Health.

[25] Allcock JA, Zhuang M, Li S, Zhao X (2024). Landscape of digital technologies used in the national health service in england: content analysis. JMIR Form Res.

[26] Willemsen RF, Aardoom JJ, van der Galiën OP, van de Vijver S, Chavannes N, Versluis A (2024). A digital platform to support communication and organization in the general practice: evaluation of healthcare usage and costs using claims data of a health insurer. Int J Med Inform.

[27] Leighton C, Joseph-Williams N, Porter A, Edwards A, Cooper A (2025). A theory-based analysis of the implementation of online asynchronous telemedicine platforms into primary care practices using Normalisation Process Theory. BMC Prim Care.

