# Supplementary material for: Digital Information Sharing Before Consultations in General Practice: Protocol for a Scoping Review
Source: JMIR Res Protoc. 2025 Dec 3;14:e82649. doi: 10.2196/82649 (PMC12712561; doi:10.2196/82649)
Supplement: Multimedia Appendix 2 [file resprot_v14i1e82649_app2.docx]

Digital information sharing before consultations in primary and community healthcare settings

| Medline via Ovid | 2694 |
| --- | --- |
| Embase via Ovid | 2640 |
| Cochrane Library | 574 |
| CINAHL via EbscoHost | 1083 |
| Total | 6991 |
| Total after deduplication | 4536 |

Searches run 12^th^ May 2025

Date limit of 2021 to present

## Medline

Ovid MEDLINE(R) and Epub Ahead of Print, In-Process, In-Data-Review & Other Non-Indexed Citations, Daily and Versions <1946 to May 09, 2025>

<https://ovidsp.ovid.com/athens/ovidweb.cgi?T=JS&NEWS=N&PAGE=main&SHAREDSEARCHID=2Ty8a83KJr6AV5wF5oRXxeYASuYIb4KtOnGuIa0KQD0vO2qCkKkaD3XFgMymJHyhG>

1 primary health care/ or (primary care or primary medical care).ti,ab. or primary health*.ti,ab,kw,kf. or general practice.ti,ab,kw,kf. or general practice/ or family practice/ or (family practice or family medicine).ti,ab,kw,kf. or (general practitioner* or gp or general physician$).ti,ab,kw,kf. or (family physician$ or family doctor* or family practitioner*).ti,ab,kw,kf. or physicians, family/ or community health services/ or (communit* adj3 health*).ti,ab,kw,kf. 490206

2 ("AccuRx" or "eConsult" or "AskMyGP" or "teledoc" or "webGP" or "PATCHS" or "klinik access" or "Q doctor" or "livi" or "healthinote" or "mychart" or "maple" or "doctolib" or "KRY" or "MDLIVE" or "amwell" or "online consultation" or "pre-consultation" or remote consult* or teleconsult* or "virtual consult*" or electronic consult* or (asynchronous adj3 (telehealth* or tele-health* or ehealth or e-health or electronic health or consult*)) or (text adj2 (consult* or appoint* or messg*)) or (email adj2 (consult* or appoint*))).ti,ab,kw,kf. or exp remote consultation/ or exp telemedicine/ 59201

3 1 and 2 6393

4 limit 3 to yr="2021 -Current" 2694

## Embase

Embase <1974 to 2025 May 09>

<https://ovidsp.ovid.com/athens/ovidweb.cgi?T=JS&NEWS=N&PAGE=main&SHAREDSEARCHID=56dHIijG1tWkcOZ5pTwGsyoxSiWaoZdurHlCRhq0OGHaxhC22kpTgbcmHVjyqW8Q0>

1 (primary care or primary medical care or primary health* or general practice or (family practice or family medicine) or (general practitioner* or gp or general physician$) or (family physician$ or family doctor* or family practitioner*) or (communit* adj3 health*)).ti,ab. 529386

2 exp *primary medical care/ 43254

3 exp *general practice/ 41659

4 exp *general practitioner/ 30403

5 exp *community care/ 62353

6 or/1-5 600973

7 ("AccuRx" or "eConsult" or "AskMyGP" or "teledoc" or "webGP" or "PATCHS" or "klinik access" or "Q doctor" or "livi" or "healthinote" or "mychart" or "maple" or "doctolib" or "KRY" or "MDLIVE" or "amwell" or "online consultation" or "pre-consultation" or remote consult* or teleconsult* or "virtual consult*" or electronic consult* or (asynchronous adj3 (telehealth* or tele-health* or ehealth or e-health or electronic health or consult*)) or (text adj2 (consult* or appoint* or messg*)) or (email adj2 (consult* or appoint*))).ti,ab. 11968

8 exp *teleconsultation/ 5280

9 exp *telemedicine/ 42441

10 or/7-9 51197

11 6 and 10 5591

12 limit 11 to yr="2021 -Current" 2640

## CINAHL

| **#** | **Query** | **Limiters/Expanders** | **Last Run Via** | **Results** |
| --- | --- | --- | --- | --- |
| S1 | XB (("primary care" or "primary medical care" or "primary health*" or "general practice" or ("family practice" or "family medicine") or ("general practitioner*" or gp or "general physician*") or ("family physician*" or "family doctor*" or "family practitioner*") or (communit* n3 health*))) | Expanders - Apply equivalent subjects  Search modes - Proximity | Interface - EBSCOhost Research Databases  Search Screen - Advanced Search  Database - CINAHL | 192,704 |
| S2 | (MH "Primary Health Care") OR (MH "Family Practice") OR (MH "Physicians, Family") OR (MH "Community Medicine") | Expanders - Apply equivalent subjects  Search modes - Proximity | Interface - EBSCOhost Research Databases  Search Screen - Advanced Search  Database - CINAHL | 116,736 |
| S3 | S1 OR S2 | Expanders - Apply equivalent subjects  Search modes - Proximity | Interface - EBSCOhost Research Databases  Search Screen - Advanced Search  Database - CINAHL | 234,135 |
| S4 | XB (("AccuRx" or "eConsult" or "AskMyGP" or "teledoc" or "webGP" or "PATCHS" or "klinik access" or "Q doctor" or "livi" or "healthinote" or "mychart" or "maple" or "doctolib" or "KRY" or "MDLIVE" or "amwell" or "online consultation" or "pre-consultation" or "remote consult*" or teleconsult* or "virtual consult*" or "electronic consult*" or (asynchronous n3 (telehealth* or tele-health* or ehealth or e-health or "electronic health" or consult*)) or (text n2 (consult* or appoint* or messg*)) or (email n2 (consult* or appoint*)))) | Expanders - Apply equivalent subjects  Search modes - Proximity | Interface - EBSCOhost Research Databases  Search Screen - Advanced Search  Database - CINAHL | 2,396 |
| S5 | (MH "Remote Consultation") OR (MH "Telemedicine+") | Expanders - Apply equivalent subjects  Search modes - Proximity | Interface - EBSCOhost Research Databases  Search Screen - Advanced Search  Database - CINAHL | 29,976 |
| S6 | S4 OR S5 | Expanders - Apply equivalent subjects  Search modes - Proximity | Interface - EBSCOhost Research Databases  Search Screen - Advanced Search  Database - CINAHL | 31,295 |
| S7 | S3 AND S6 | Expanders - Apply equivalent subjects  Search modes - Proximity | Interface - EBSCOhost Research Databases  Search Screen - Advanced Search  Database - CINAHL | 2,969 |
| S8 | S3 AND S6 | Limiters - Publication Date: 20210101-20251231  Expanders - Apply equivalent subjects  Search modes - Proximity | Interface - EBSCOhost Research Databases  Search Screen - Advanced Search  Database - CINAHL | 1,083 |

## Cochrane

ID Search Hits

#1 ("primary care" or "primary medical care" or (primary NEXT health*) or "general practice" or "family practice" or "family medicine" or (general NEXT practitioner*) or gp or (general NEXT physician*) or (family NEXT physician*) or (family NEXT doctor*) or (family NEXT practitioner*) or (communit* NEAR health*)):ti,ab,kw 55501

#2 MeSH descriptor: [Family Practice] explode all trees 2343

#3 MeSH descriptor: [Primary Health Care] explode all trees 11903

#4 MeSH descriptor: [Physicians, Family] explode all trees 541

#5 MeSH descriptor: [Community Health Services] explode all trees 19411

#6 #1 or #2 or #3 or #4 or #5 73637

#7 ("AccuRx" or "eConsult" or "AskMyGP" or "teledoc" or "webGP" or "PATCHS" or "klinik access" or "Q doctor" or "livi" or "healthinote" or "mychart" or "maple" or "doctolib" or "KRY" or "MDLIVE" or "amwell" or "online consultation" or "pre-consultation" or (remote NEXT consult*) or teleconsult* or (virtual NEXT consult*) or (electronic NEXT consult*) or (asynchronous NEAR (telehealth* or tele-health* or ehealth or e-health or "electronic health" or consult*)) or (text NEAR (consult* or appoint* or messg*)) or (email NEAR (consult* or appoint*)) ):ti,ab,kw 2154

#8 MeSH descriptor: [Remote Consultation] explode all trees 480

#9 MeSH descriptor: [Telemedicine] explode all trees 5312

#10 #7 OR #8 OR #9 6884

#11 #6 AND #10 with Cochrane Library publication date Between Jan 2021 and Jan 2025 527

#12 #6 AND #10 with Publication Year from 2021 to 2025, in Trials 530

#13 #11 OR #12 574
